# Lessons from intensified surveillance of viral hepatitis A, Israel, 2017 and 2018

**DOI:** 10.2807/1560-7917.ES.2021.26.6.2000001

**Published:** 2021-02-11

**Authors:** Yael Gozlan, Itay Bar-Or, Hadar Volnowitz, Efrat Asulin, Rivka Rich, Emilia Anis, Yonat Shemer, Moran Szwarcwort Cohen, Etti Levy Dahary, Licita Schreiber, Ilana Goldiner, Orit Rozenberg, Orit Picard, Michal Savion, Inbal Fuchs, Ella Mendelson, Orna Mor

**Affiliations:** 1 Central Virology Laboratory, Ministry Of Health, Sheba Medical Center, Ramat-Gan, Israel; 2 Public Health Services, Ministry Of Health, Jerusalem, Israel; 3 Hebrew University Hadassah Braun School of Public Health and Community Medicine, Jerusalem, Israel; 4 Virology Laboratory, Soroka University Medical Center, Beer-Sheva, Israel; 5 Virology Laboratory, Rambam Health Care Campus, Haifa, Israel; 6 Meuhedet Health Services, Lod, Israel; 7 Maccabi Health Services, Mega Laboratory, Rehovot, Israel; 8 Clinical Biochemistry Laboratory, Tel-Aviv Sourasky Medical Center, Tel-Aviv, Israel; 9 Immunological Laboratory, Emek Medical Center, Afula, Israel; 10 Gastroenterology Laboratory, Sheba Medical Center, Ramat Gan, Israel; 11 Tel-Aviv District Health Office, Ministry Of Health, Tel Aviv, Israel; 12 Clalit Health Services, Southern district Beer Sheva, Israel; 13 School Of Public Health, Tel Aviv University, Tel Aviv, Israel; 14 These authors contributed equally to this article

**Keywords:** hepatits A, HAV, hepatitis A outbreak, genotyping, sewage sampling, surveillance framework, hepatitis A vaccinated population

## Abstract

**Introduction:**

Universal vaccination of toddlers has led to very low hepatitis A (HAV) endemicity in Israel. However, sporadic outbreaks still occur, necessitating better surveillance.

**Aim:**

To implement a comprehensive HAV surveillance programme.

**Methods:**

In 2017 and 2018, sera from suspected HAV cases that tested positive for anti-HAV IgM antibodies were transferred to the Central Virology Laboratory (CVL) for molecular confirmation and genotyping. Sewage samples were collected in Israel and Palestine* and were molecularly analysed. All molecular (CVL), epidemiological (District Health Offices and Epidemiological Division) and clinical (treating physicians) data were combined and concordantly assessed.

**Results:**

Overall, 146 cases (78 in 2017 and 68 in 2018, median age 34 years, 102 male) and 240 sewage samples were studied. Most cases (96%) were unvaccinated. In 2017, 89% of cases were male, 45% of whom were men who have sex with men (MSM). In 2018, 49% were male, but only 3% of them were MSM (p < 0.01). In 2017, 82% of cases and 63% of sewage samples were genotype 1A, phylogenetically associated with a global MSM-HAV outbreak. In 2018, 80% of cases and 71% of sewage samples were genotype 1B, related to the endemic strain previously identified in Israel and Palestine*. Environmental analysis revealed clustering of sewage and cases’ sequences, and country-wide circulation of HAV.

**Conclusions:**

Molecular confirmation of HAV infection in cases and analysis of environmental samples, combined with clinical and epidemiological investigation, may improve HAV surveillance. Sequence-based typing of both clinical and sewage-derived samples could assist in understanding viral circulation.

## Introduction

Hepatitis A virus (HAV) is the most common cause of viral hepatitis worldwide. It is transmitted primarily via the faecal/oral route by ingestion of contaminated food or water, or through direct contact with an infectious person. Infection does not result in chronic liver disease, but can cause debilitating symptoms and lead to acute liver failure, which is associated with high mortality [1]. In low-income countries, HAV remains highly endemic, but improvements in water and sanitation systems are reducing transmission rates. In many European countries, as well as in the United States (US) and other countries with effective sanitation and hygiene practices, HAV endemicity is low and the anti-HAV vaccine—which was, for example, approved by the US Food and Drug Administration in 1995 [2]—is most often recommended to individuals at increased risk of infection and to cases’ contacts [3]. In such countries, non-vaccinated adults remain susceptible to HAV and in recent years, outbreaks have become more frequent [4].

Visiting an endemic country 2 to 6 weeks before the onset of symptoms is one of the main risks for acquiring infection with HAV. In a study reporting causes of HAV infection in 13 European countries, 27.8% of reported hepatitis A cases between 2009 and 2015 were travel associated [5]. HAV outbreaks among MSM have been reported in European [6] and other countries [7-9], and—to a much lesser degree—in association with consumption of imported HAV-contaminated food [10].

In Israel, a country with a universal anti-HAV childhood vaccination programme since 1999—wherein children are vaccinated with two doses, at 1.5 and 2 years—two outbreaks were reported in the last 10 years. The first occurred in 2012 and 2013 among an unvaccinated homeless population in the Tel Aviv district [11]. The second, which started towards the end of 2016, was also mainly restricted to the Tel Aviv district. Unlike the previous outbreak, the latter involved non-vaccinated MSM, among whom HAV was primarily transmitted via sexual contacts, similar to the MSM outbreaks in Europe [12].

The HAV genome is a 7.5 kb ssRNA, non-enveloped, acid and heat stable picornavirus. The most common genotypes in humans are 1 and 3, which are divided into 2 subgenotypes, A and B, with distinctive geographical and risk group distribution. Genotype 3A has been reported in India and the Central Asian Republics of the former Soviet Union [13], while specific strains of genotype 1A were recently linked to a global HAV outbreak among MSM [6-9].

Diagnosis of acute hepatitis A is usually based on clinical signs consistent with acute viral hepatitis (e.g. fever, headache, malaise, anorexia, nausea, vomiting, diarrhoea, abdominal pain, abnormal liver function tests) together with the detection of anti-HAV IgM antibodies in the blood. Molecular analysis is not globally required for confirmation of HAV diagnosis. In the European Union, hepatitis A is a notifiable disease; although repeat testing or molecular confirmation are not always performed, harmonisation of HAV typing practices was initiated in 2014 [14]. A recent report demonstrates that not all European countries perform centralised sample collection, making it challenging to compare HAV sequences in cases of cross-border HAV outbreaks [15].

Environmental surveillance for polio, a vaccine-preventable enteric virus, has demonstrated the ability to detect silent introduction of wild type poliovirus in the absence of clinical cases [16,17]. In Italy, surveillance of HAV in urban sewage demonstrated consistency with the classification of Italy as a low/intermediate endemic country [18].

The most recent HAV outbreak in Israel raised the need for better national HAV surveillance. Until this outbreak, diagnosis of HAV was based on the clinical picture of acute hepatitis and anti-HAV IgM positivity. Since 2017, molecular confirmation of HAV serology and sampling from major sewage treatment facilities throughout Israel were introduced. We describe the results obtained by this intensified surveillance programme in 2017 and 2018, as well as the lessons learnt.

## Methods

### Study design, clinical samples and data collection

This observational study was based on data collected between January 2017 and December 2018.

In Israel, hepatitis A cases are reported to the Ministry of Health (MoH), as mandated by the State Public Health Law. Clinicians inform the district health offices (DHS) of a suspected case. The DHS conducts epidemiological investigations using a structured questionnaire and notifies the epidemiological division (ED) of public health services (PHS), which holds a database of all HAV infection cases (HAV registry).

Prior to this study, IgM-positive serology was the only result directly related to HAV that was used to confirm HAV infection. Since January 2017, PHS requested that molecular confirmation be performed at the viral hepatitis reference centre in the Central Virology Laboratory (CVL), at Sheba Medical Center, Ramat Gan. District laboratories identifying blood samples positive for anti-HAV IgM antibodies (> 1.2 s/co, Architect, Abbott, US) or with grey zone IgM results (0.8–1.2 s/co) were requested to transfer the remains of these sera samples to the CVL for molecular confirmation. When serum remains were not available for molecular assessment, HAV status was determined based on the anti-HAV IgM results, the reports from the treating physicians and the epidemiological investigation provided by the DHS.

Data was continuously collected and updated in the national HAV database. Chi-square or Fisher exact tests were used for statistical analysis. P value was considered significant if it was lower than 0.01.

### Case definition

In this study, all cases that met the clinical HAV criteria, i.e. IgM anti-HAV positive and HAV-RNA positive by PCR, were considered HAV confirmed. IgM positive cases meeting clinical criteria that could not be confirmed molecularly because of a lack of samples were considered possible cases. However, if contact (e.g. household, travel to an endemic country, sexual) with a laboratory-confirmed HAV case 15 to 50 days before onset of symptoms could be established, these cases were considered HAV confirmed. Individuals meeting clinical criteria but lacking laboratory confirmation were excluded.

### Sewage sample collection

Sewage samples were collected monthly, as part of the routine supplemental polio surveillance programme, from four major districts in Israel (Jerusalem, Tel Aviv, Haifa and Beer-Sheva) and from Tsfat. From Palestine* (including Gaza, Nablus and Hebron), samples were collected every 3–4 months. Each of the 500 mL sewage samples collected was concentrated to 3–5 mL, as previously described [11].

### Hepatitis A virus molecular confirmation

Total nucleic acids were extracted from 400 µL of serum using NucliSENS easyMag (bioMérieux, Marcy-l'Étoile, France). Total nucleic acids extractions in concentrated sewage were preliminarily prepared with omnicleave endonuclease (epicenter, Lucigen, Middleton, US) to reduce the load of free nucleic acids in the samples. Then 300 µL concentrated sewage were extracted using NucliSENS easyMag (bioMérieux). RealStar HAV real-time PCR assay (version 1.0, Altona diagnostics, Hamburg, Germany) or in-house PCR assay (described below for sequencing) were used for molecular confirmation of HAV infection.

### Hepatitis A virus sequencing and genotyping

All real-time PCR–positive samples were genotyped using PCR product that covered a 460-nt fragment located within the VP1/P2A region according to the HAVNET unified typing protocol [14]. Sequencing was performed as previously described [12]. HAV genotype was determined using the Hepatitis A Virus Automated Genotyping Tool [19].

### Phylogenetic analysis

HAV nt sequences were cured and aligned to the M14707 reference sequence using the Open-gene system (Siemens, Malvern, US). Phylogenetic analysis was conducted using a neighbour-joining algorithm in MEGA, version 6, with 1,000 replicates for bootstrap testing. Representative reference nt sequences for various genotypes included in the phylogenetic analysis were the MH577314.1 for US genotype 1A, KX22869.1 for Egypt genotype 1B, AF314208.1 for China genotype 1A, X83302.1 for Italy genotype 1A, V16–25801 VP1 for Germany genotype 1A, RIVM europride 2016 1A for Europride genotype 1A, MN062164.1 for US genotype 1A, VRD521 521 1A for England genotype 1A, MGO49743.1 for São Paulo genotype 1A and DQ991030.1 for India genotype 3A, all from GenBank.

All sequences were deposited in the National Center for Biotechnology Information database, accession numbers MT570190 - MT570349.

### Ethical statement

This study was approved by the Sheba Medical Centre Institutional Review Board (number SNC-54819).

## Results

### Molecular confirmation of anti-HAV IgM positive results

Sera remains of samples reactive for anti-HAV IgM antibodies (n = 193) were tested for the presence of HAV RNA. Molecular analysis revealed that all grey zone samples (n = 41) were HAV RNA negative. Furthermore, no clinical signs of HAV infection were reported by the treating physicians; therefore, these cases were excluded from further analysis. Moreover, six anti-HAV IgM positive cases (above 1.2 s/co; four in 2017 and two in 2018) were not confirmed molecularly. In these cases, epidemiological investigation could not support HAV infection; therefore, they were excluded from the analysis. All other cases with available sera for molecular analysis were confirmed as described below and HAV was sequenced and genotyped.

### Clinical hepatitis A virus infection cases

We identified 146 cases of HAV infection: 78 in 2017 and 68 in 2018. Table 1 summarises the data for all HAV infection cases. The median age of all cases was 34 years. Only five (3.4%) cases were > 65 years old and 10 (6.8%) were < 20 years old, all born after 1999, when the vaccination programme was implemented.

**Table 1:** Characteristics of hepatitis A virus infection cases, Israel, 2017–2018 (n = 146)

Characteristics	2017			2018			p value
	N	All	%	N	All	%	
Male	69	77	89	33	68	49	< 0.01
Hospitalised	45	78	57	32	60	53	0.609
MSM^a^	31	69	45	1	3	33	< 0.01
Hepatitis A vaccination^b^	4	53	8	1	60	2	0.185
**Residence**							
Tel Aviv district	37	78	47	12	68	18	< 0.01
Jerusalem district	7		9	15		22	0.023
Haifa district	8		10	7		10	0.985
Beer-Sheva district	12		15	12		18	0.713
Other or unknown regions	14		18	22		32	0.044
**HAV genotype**							
1A	41	50	82	6	47	13	< 0.01
1B	8		16	38		80	< 0.01
3	1		2	3		6	0.278

HAV: hepatitis A virus; MSM: men who have sex with men.

^a^ Self-identified as MSM.

^b^ Cases who received post-exposure vaccination were considered as vaccinated.

Family contacts accounted for 2.6% (2/78) of the cases in 2017, while in 2018 five family/work contacts accounted for 19.1% (13/68) of the HAV infections. Hospitalisation rates were relatively similar between the studied years: 57% in 2017 and 53% in 2018. However, in 2017, 89% of the cases were male and 45% self-identified as MSM, while in 2018 only 49% were male and only 3% self-identified as MSM (p < 0.01). While in 2017 most of the HAV cases (47%) were from the Tel Aviv district, in 2018 only 18% were from this area (p < 0.01). On the other hand, the proportion of cases from the Jerusalem district was significantly higher in 2018 (22%) compared with 2017 (9%; p = 0.023).

Several operational steps were taken in 2017 to curb the HAV outbreak among MSM. Information was disseminated on social media, leaflets explaining HAV transmission were distributed, free vaccination was offered and HAV cases’ household and sexual contacts were sought.

Most (96%) of the cases in 2017 and 2018, including all those < 20 years old, were not vaccinated. Epidemiological investigation revealed that three of those considered to have been vaccinated received the anti-HAV vaccine post-exposure. Date of vaccination was not available for the other two individuals.

Samples from 50 cases in 2017 and 47 cases in 2018 were available for molecular analysis. HAV genotype 1A was found in 41 of 50 cases in 2017 and only in 6 of 47 cases in 2018 (p < 0.01). Moreover, while in 2017 genotype 1B was found in eight of 50 cases, in 2018, 38 of 47 cases were infected with genotype 1B (p < 0.01). Only a single case in 2017 and three cases in 2018 were found infected with genotype 3; all were travellers or had direct contact with travellers returning from endemic countries. Importation of genotype 1 was recorded for 40% of cases in 2017 and for 17% [8] of cases in 2018; 96% (27/28) of these had travelled to European countries and one to the US.

### Environmental surveillance

A total of 240 sewage samples were assessed: 122 in 2017 and 118 in 2018 (Table 2). Nineteen of the samples (nine in 2017 and 10 in 2018) were collected from Palestine*. Overall, 45% (55/122) of sewage samples in 2017 and 27% (32/118) in 2018 were HAV positive (p < 0.05). Reduction in the prevalence of HAV-positive sewage samples was mainly observed in the Tel Aviv district (25/41, 61% in 2017 vs 8/28, 21% in 2018; p < 0.01). Of note is the high percentage of HAV-positive sewage samples from the Haifa district in both 2017 (83%) and 2018 (72%), although the Haifa district was not the epicentre of any outbreak in these years. Genotype could be determined for 77% (67/87) of the HAV-positive sewage samples. In 2017, the more frequent subgenotype was 1A (31/55), while in 2018 subgenotype 1B was dominant (24/32; p = 0.108). HAV genotype 3 was not found in any of the sewage samples. All successfully sequenced sewage samples collected in Palestine* (9/19) were classified as subgenotype 1B.

**Table 2:** Characteristics of sewage and clinical samples by district, (sub)genotype and year of collection, Israel and Palestine*, 2017–2018

District	Sewage samples			Case samples		
	HAV (sub)genotype	2017 (n)	2018 (n)	HAV (sub)genotype	2017 (n)	2018 (n)
Jerusalem	1A	3	0	1A	1	0
	1B	0	8	1B	0	4
	UD	2	1	3	0	0
	All positives	5	9	Samples sequenced	1	4
	All samples	36	36	All cases recorded	7	12
Tel Aviv	1A	20	2	1A	25	2
	1B	0	6	1B	2	13
	UD	5	0	3	1	0
	All positives	25	8	Samples sequenced	28	15
	All samples	41	38	All cases recorded	37	15
Haifa	1A	4	0	1A	1	2
	1B	3	4	1B	2	2
	UD	3	4	3	0	2
	All positives	10	8	Samples sequenced	3	6
	All samples	12	11	All cases recorded	8	7
Beer-Sheva	1A	4	0	1A	1	0
	1B	0	1	1B	0	2
	UD	1	1	3	0	0
	All positives	5	2	Samples sequenced	1	2
	All samples	12	12	All cases recorded	1	3
Tzfat	1A	0	0	1A	0	0
	1B	2	1	1B	0	0
	UD	2	0	3	0	0
	All positives	4	1	Samples sequenced	0	0
	All samples	12	11	All cases recorded	0	0
Other	1A	NA	NA	1A	13	2
	1B	NA	NA	1B	4	17
	UD	NA	NA	3	0	1
	All samples	NA	NA	Samples sequenced	17	20
	All cases recorded	NA	NA	All cases recorded	17	20
Palestine*	1A	0	0	1A	NA	NA
	1B	5	4	1B	NA	NA
	UD	1	0	3	NA	NA
	All positives	6	4	Samples sequenced	NA	NA
	All samples	9	10	All cases recorded	NA	NA

HAV: hepatitis A virus; NA: not available; UD: undetermined genotype (HAV-positive samples).

All sewage samples and cases identified in 2017 and 2018 are presented.

* This designation shall not be construed as recognition of a State of Palestine and is without prejudice to the individual positions of the European Union Member States on this issue.

### Comparison between clinical cases and environmental results

**Figure 1:**
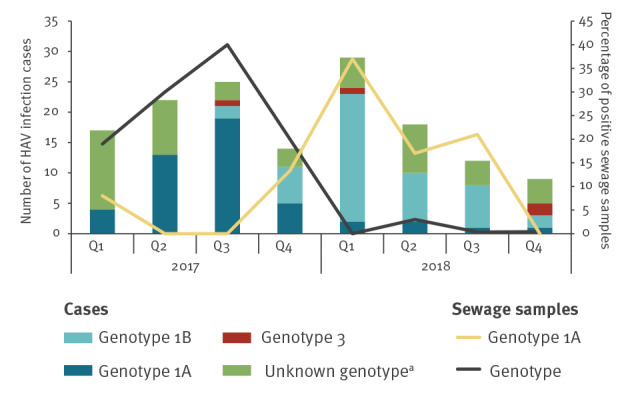
Environmental and clinical hepatitis A virus surveillance, Israel, 2017–2018

HAV: hepatitis A virus; Q: quarter.

^a^ Those indicated as ‘Unknown genotype’ were cases considered as HAV positive, for whom blood samples were not available for molecular confirmation.

The segregation of HAV-positive samples by (sub)genotype (1A, 1B, 3 or undetermined), geographical location and year of collection (Table 2) was highly similar between environmental samples and clinical cases. This observation is supported by the similarity between the timely pattern of HAV cases’ diagnosis, detection of positive sewage samples and the type of viral genotypes that were identified throughout 2017 and 2018 (Figure 1). While information on HAV cases in Palestine* is not available to the PHS (Table 2), about half of sewage samples collected from facilities in Palestine* in 2017 and 2018 (10/19) were positive for subgenotype 1B.

### Phylogenetic analysis

**Figure 2:**
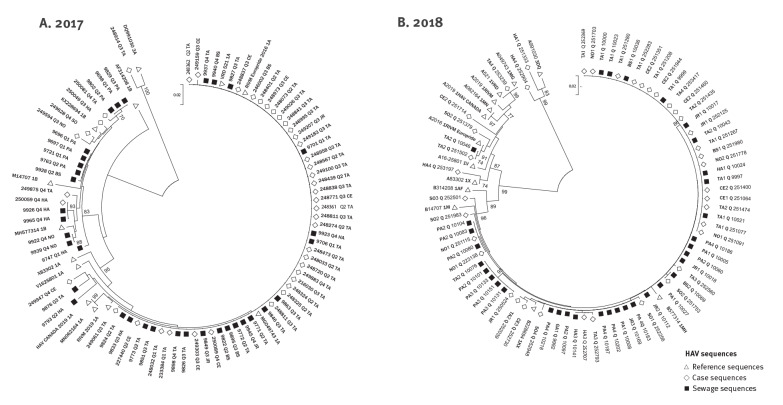
Phylogenetic analysis of clinical and environmental hepatitis A virus sequences, Israel and Palestine*, 2017–2018

BS: Beer-Sheva; CE: central; HA: Haifa; JR: Jerusalem; NO: north; PA: Palestine*; Q: quarter; SO: south; TA: Tel Aviv.

Phylogenetic analysis revealed clustering of sewage and clinical samples both in 2017 and in 2018 (Figure 2). In 2017, the large cluster of subgenotype 1A—which included both clinical and sewage sequences—aligned with both of the strains that were reported in MSM outbreaks in Europe: the RIVM europride 2016 1A and the VRD 521 1A [6]. Clusters of 1B in both 2017 and 2018 aligned with the M14707 1B strain that was previously reported to be circulating in Israel [11]. Also, HAV 1B sequences from all environmental samples clustered together with 1B sequences identified in cases from different regions in the country, as well as with those previously reported to be circulating in Israel [11].

Neighbour-joining tree of sequences of the VP1/P2A region (A- 428bp, B- 461bp) of HAV isolates. Several reference strains (denoted by GeneBank accession number) were also included. Numbers at the nodes indicate bootstrap values. Each sequence is presented with its sample number, time of diagnosis (yearly quarter, Q1–Q4) and location. Distance scale (0.02) denotes 2% difference between any two sequences.

* This designation shall not be construed as recognition of a State of Palestine and is without prejudice to the individual positions of the European Union Member States on this issue.

## Proposed model for hepatitis A virus surveillance

**Figure 3:**
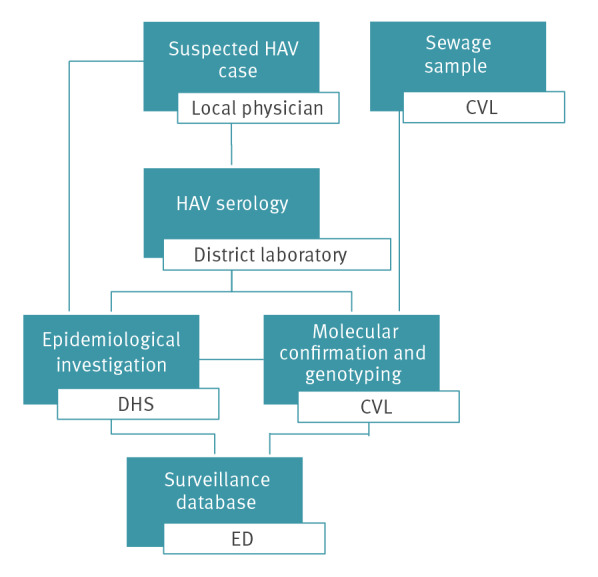
Proposed model for hepatitis A virus surveillance in countries with childhood vaccination programmes

CVL: Central Virology Laboratory, Ministry of health; DHS: district health offices; ED: epidemiological division of public health services at the Ministry of health; HAV: hepatitis A virus.

Based on the findings presented, a model that outlines the diagnostic steps and the personnel required for efficient surveillance in low endemic countries is hereby suggested (Figure 3).

## Discussion

This study demonstrates the added value of molecular and sequencing analysis of both clinical and environmental samples for the surveillance of an enteric virus like HAV.

In Israel, HAV incidence was below 0.7 cases per 100,000 population in the years prior to the study period (54 cases in 2014, 53 in 2015, 51 in 2016). Higher numbers of cases were reported in 2017 (n = 78) and 2018 (n = 68). In 2017, our data showed that the HAV 1A outbreak among MSM in Israel accounted for this higher incidence. In contrast, the number of HAV 1B cases in 2017 was much lower than in 2018, when 1B was the dominant subgenotype. Under-reporting of sporadic cases, mainly in 2017—when this enhanced surveillance system and data sharing between the CVL and ED had only recently been established, with most efforts directed towards stopping the subgenotype 1A outbreak—may account for this low 1B incidence. Epidemiological investigation further revealed that the few 1B cases in 2017 and those in 2018 were sporadic, some of which were transmissions within unvaccinated families. Indeed, in 2019, when the aforementioned collaborative workflow continued to operate, more cases (n = 86) were reported and 88.6% of the molecularly confirmed cases were found to be subgenotype 1B (data not shown). Such synergistic impact of collaboration between reporting systems was previously demonstrated in Catalonia, where combining the Statutory Disease Reports and Microbiological Reporting Systems improved the efficiency of HAV case detection [20].

Another aspect of the presented framework is the value of molecular confirmation of HAV infection. Specificity is a known obstacle in serology. For example, it is known that older HAV cases can have a non-specific increase of anti-HAV IgM levels. Here, molecular analysis confirmed all IgM results of the few cases > 65 years old. Molecular analysis further revealed that two individuals with positive anti-HAV IgM sera were HAV-RNA negative. Follow-up epidemiological analysis corroborated these findings. Consequently, these individuals were not analysed here. Indeed, the US Centres for Disease Control and Prevention have reported that in 2017 and 2018, 8–10% of IgM positive samples were not confirmed by RNA analysis [21]. In our study, several of the IgM positive cases (28 in 2017 and 21 in 2018) were not molecularly tested due to logistical issues or lack of appropriate serum samples. Lack of molecular confirmation of all clinical cases is one of the limitations of our study. However, epidemiological investigation revealed that the vast majority of these cases, described herein, had been exposed to HAV (e.g. via importation, food contamination, sexual contacts), therefore indicating that all had genuine HAV infections.

HAV genotyping and phylogenetic analysis assists in transmission analyses during an outbreak investigation. In 2017, the majority of cases and many of the sewage samples were from the Tel Aviv district and were classified as subgenotype 1A, which was in agreement with the reports on the specific MSM HAV strains reported in the outbreaks in Europe in 2016 and 2017 [6]. These results, together with the demographic data and epidemiological investigation, indicated that the source of the outbreak in Israel was indeed European. In 2018, when the MSM outbreak had been controlled, the dominant subgenotype identified in cases from Tel Aviv—as well as from other districts—was 1B (and not 1A), which clustered with 1B sequences found previously in Israel [11].

The impact of environmental sampling for studying the circulation of an enteric virus is also demonstrated in this study. The most abundant strains of HAV subgenotypes (1A and 1B) identified in sewage and in clinical cases clustered together. Bisseux et al., in France, have also shown that environmental surveillance detected viral sequences that were genetically similar to those reported in clinical specimens [22]. Moreover, the correlation of time, place and prevalence of different HAV genotypes between the cases’ and the environmental samples suggests that the latter can provide vital information on the circulation of enteric viruses such as HAV in countries where clinical cases are rare or under-reported. Detection of pathogenic viruses in sewage has also been shown to provide an early warning of HAV and norovirus outbreaks [23].

Israel experienced silent circulation of wild poliovirus type 1 in 2013 and 2014, detected through environmental surveillance of the sewage system with no cases of acute flaccid paralysis [24]. In this study, about half of the sewage samples in Palestine* were HAV positive and 1B was the only (sub)genotype identified, suggesting continuous circulation of this strain within the local population. Moreover, the Palestine*-derived 1B sequences clustered with all 1B sequences identified in this study, as well as those previously reported to be circulating in Israel and Palestine* [11]. Although clinical cases in Palestine* are not reported to the Israeli PHS, our results suggest that HAV 1B is endemic in Palestine* and in our region, and strains need continuous monitoring.

There are several limitations to environmental surveillance. One of the drawbacks is its rather low sensitivity. Even if sewage samples are found to be HAV negative, members of the population might still be infected and secrete enteric viruses [25]. It has been estimated that in industrialised communities, ca 1% of the population need to excrete the virus in order for it to be detected by sewage surveillance [17]. Indeed, genotype 3, which was identified in only a few cases each year, was not detected in sewage, probably due its very low prevalence in the local population. Functional analysis was also not included in this study. Functional data, obtained by assessing the cytopathic effect of the environmental samples in tissue culture, would have ascertained the potential infectivity of the sewage-derived HAV sequences. However, HAV rarely grows in cell culture and requires several weeks to months in culture before it can be detected [26]. Another drawback of using a molecular approach to the analysis of sewage samples was raised in a study from Italy, where the variants restricted to detections in sewage samples were related to strains involved in past outbreaks [27]. Here, the environmental and clinical data showed the same trend, arguing against such a possibility. The cost-effectiveness of sewage surveillance, which was not investigated here, should also be considered, especially in countries without an established environmental sampling programme. Lastly, current sewage surveillance methodology does not cover all regions in Israel. For example, samples from facilities around the centre (e.g Hasharon and Ashdod) are not regularly collected. Expanding sewage sampling to other areas in the country would be beneficial.

### Conclusion

In regions such as Israel that have low HAV incidence, molecular confirmation and phylogenetic analysis performed simultaneously with clinical and epidemiological investigation of all suspected cases could hasten identification of an outbreak. Once an outbreak is identified, measures to contain it can be implemented instantly. Ongoing analysis of environmental samples could improve the overall quality of surveillance in countries with national HAV vaccination programmes.

## Note

**This designation shall not be construed as recognition of a State of Palestine and is without prejudice to the individual positions of the European Union Member States on this issue.*
